# Relationship Between Plant Diversity and Soil Environment in Karst Urban Remnant Mountains: A Comparative Analysis of Two Types

**DOI:** 10.1002/ece3.71174

**Published:** 2025-03-19

**Authors:** Shujun Liu, Zhijie Wang, Lifei Yu, Gilbert Kumilamba, Gang Xie

**Affiliations:** ^1^ Key Laboratory of Plant Resource Conservation and Germplasm Innovation in Mountainous Region (Ministry of Education), College of Life Sciences/Institute of Agro‐Bioengineering Guizhou University Guiyang Guizhou China; ^2^ Institute of Guizhou Mountainous Resources Guiyang Guizhou China

**Keywords:** karst urban remnant natural mountains, plant diversity, soil environment factors, urban remnant mountain parks

## Abstract

Understanding the characteristics of plant diversity and its relationship with the soil environment in urban remnant habitats before and after their transformation into parks is of great significance for strengthening urban biodiversity conservation. To investigate the changes in plant diversity characteristics and their relationship with the soil environment following the transformation of urban remnant natural mountains (URNM) into urban remnant mountain parks (URMP), we conducted a study in the urban area of Guiyang City, China. We sampled 90 plots across five typical URNM and five typical URMP. Plant diversity and its relationship with soil properties were evaluated using four taxonomic diversity indices and 9 soil physicochemical properties. The results showed that URNM exhibited higher plant diversity and richer species richness compared to URMP. In URNM, plant survival conditions deteriorate with the elevation of slope position, resulting in the highest plant diversity at lower slopes and the lowest at upper slopes. However, intense human disturbances lead to the opposite pattern in URMP. Additionally, soil bulk density, total phosphorus, and total potassium (TK) were found to be higher in URMP than in URNM. C/N, C/P, and soil organic carbon were identified as the main factors influencing plant diversity in URNM, with explanatory rates of 20.1%, 15.4%, and 8.6%, respectively. In URMP, TK was the most significant factor, explaining over 55.9% of plant diversity. These findings indicate that the transformation of karst urban remnant mountains into parks leads to a simplification of plant species composition and a reduction in plant diversity. This process also alters the characteristics of soil environmental factors and their relationship with plant diversity. These changes highlight the need for careful management strategies in urban park development to mitigate biodiversity loss and maintain soil health, which are crucial for the sustainability of urban ecosystems.

## Graphical abstract

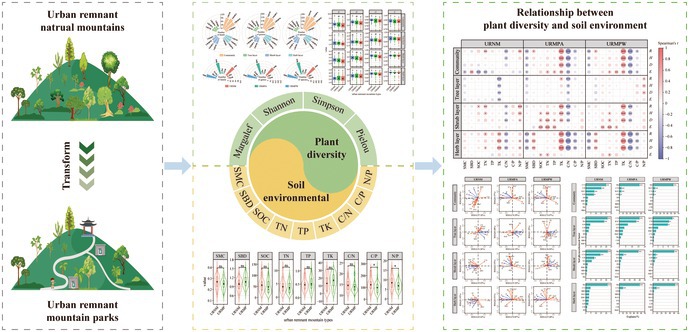

## 1 Introduction

The relationship between plant diversity and soil environment is complex, particularly in remnant habitat ecosystems (Bauer et al. 2015; Hu et al. 2021; Laliberté et al. 2014; Ma et al. 2023). This interrelationship occurs almost simultaneously, significantly influencing both local and global patterns of biodiversity, while playing a crucial role in ecosystem succession and evolution (Liu et al. 2021; Teste et al. 2017; Van Nuland et al. 2019). Soil, as the foundational medium for plant growth, is not only merely a substrate for sustaining plant life but also a key factor in shaping the structural and functional dynamics of plant ecosystems (Dorey et al. 2024; Rajakaruna 2018). In turn, plants influence soil properties through root activities and organic matter flow (Aponte et al. 2010; Li et al. 2024; Sardans and Peñuelas 2013). Hence, understanding these complex plant–soil interactions is crucial for elucidating ecosystem resilience mechanisms and developing effective strategies for ecological restoration and biodiversity conservation in changing environments.

However, the relationship between plant diversity and the soil environment is not uniform and varies across ecosystem types (Li et al. 2018; Zhang et al. 2021), geographical regions (Bhandari and Zhang 2019; Janssens et al. 1998), and under varying disturbance conditions (Wangchuk et al. 2014; Wu et al. 2021). These differences are primarily evident in the correlations between plant diversity and soil environmental factors, including carbon, nitrogen, phosphorus, and potassium, which can exhibit positive, negative, or non‐significant relationships. This highlights the importance of conducting in‐depth research on the plant–soil relationship across different ecosystems, regions, and disturbance conditions. Given this complexity, focused studies are required to explore how plant diversity interacts with soil properties in specific regions and under various environmental conditions, particularly in areas facing severe ecological degradation (Ma et al. 2020).

In this context, remnant habitats, defined as fragments of natural habitats that have survived human disturbances, have garnered increasing attention due to their critical role in maintaining biodiversity and providing essential ecosystem services (Cousins 2006; Lindgren and Cousins 2017; Mendenhall et al. 2014). However, ongoing urbanization and persistent anthropogenic disturbances have exacerbated threats to these valuable habitats. Such threats not only contribute to further reduction and fragmentation of remnant habitats but also disrupt the soil environment, posing a severe risk to the biodiversity within these ecosystems (Berthon et al. 2021; Yang et al. 2022). While much of the research on remnant habitats has focused on plant diversity and species composition (Cousins and Lindborg 2008; Polley et al. 2005; Zheng and Yang 2023), the relationship between plant diversity and soil characteristics remains underexplored. Thus, elucidating the characteristics and coupling relationship between plant diversity and the soil environment in remnant habitats is of great significance for better protecting and maintaining the biodiversity within these valuable habitats.

While remnant habitats globally face these pressures, nowhere is the urgency more acute than in Southwest China's karst regions (Yang et al. 2021; Zeng et al. 2022). Here, the unique interplay of geological fragility and rapid urbanization creates unprecedented ecological tensions (Wang et al. 2022b; Wu et al. 2024). This, in turn, provides an ideal setting for investigating the interactions between plant diversity and the soil environment. This region boasts the largest continuous expanse of karst landscapes in the world, making it one of the most ecologically vulnerable areas (Bai et al. 2023). It is also characterized by isolated peaks and peak clusters, which, due to human activities, have given rise to numerous unique karst remnant habitats, known as karst remnant mountains (Zeng et al. 2022). Furthermore, the region is one of the 36 global biodiversity hotspots, with the majority of these hotspots located within the karst remnant mountains. Consequently, the ecological significance of natural remnant mountains in karst urban areas has become increasingly prominent. Scholars have conducted a series of studies focusing on their plant diversity (Yang et al. 2022; Zeng et al. 2022), landscape patterns (Yang et al. 2021), and cooling island effects (Chen et al. 2021). Especially in recent years, the acceleration of urbanization and the continuous population growth have led to a rising demand for parks among urban residents. This has necessitated the transformation of certain natural remnant mountains in the central urban area into mountain parks, resulting in the emergence of two typical urban remnant mountain ecosystems: urban remnant natural mountains (URNM) and urban remnant mountain parks (URMP). Despite Wang et al. (2024) investigating the impacts of converting karst mountainous forests into urban parks on plant diversity patterns, whether the transformation from URNM to URMP alters plant diversity, soil environmental characteristics, and their coupling relationships remains unclear. Thus, elucidating the aforementioned issues will not only enhance our comprehension of the intricate interplay between plant diversity and the soil environment in karst urban remnant mountains but also offer a robust scientific foundation for their conservation, management, and sustainable utilization.

To address these knowledge gaps, we conducted a comparative study of two distinct karst urban remnant mountain types (five URNM, and five URMP) in a karst city. By integrating field sampling and laboratory analyses, we employed Kruskal–Wallis tests, Spearman correlation analysis, and redundancy analysis (RDA) to investigate the characteristics and relationships between plant diversity and soil environmental factors in URNM and URMP. This study aims to: (1) ascertain the plant composition, diversity characteristics, and distribution patterns of distinct types of urban remnant mountains; (2) elucidate the characteristics of soil environmental factors in various types of urban remnant mountains; (3) reveal the coupling relationship between plant diversity and soil environmental factors in different types of urban remnant mountains and identify the key soil factors influencing plant diversity. We hypothesize that the transformation of URNM into URMP will lead to a reduction in plant diversity and alter the distribution patterns of plant diversity. Additionally, soil physicochemical properties will change due to human disturbances. Ultimately, this may result in differences in the main soil physicochemical factors affecting URNM and URMP. This study will provide a scientific basis for the conservation and sustainable management of the remnant habitats in karst and similar areas.

## 2 Methods

### 2.1 Study Area

**FIGURE 1:**
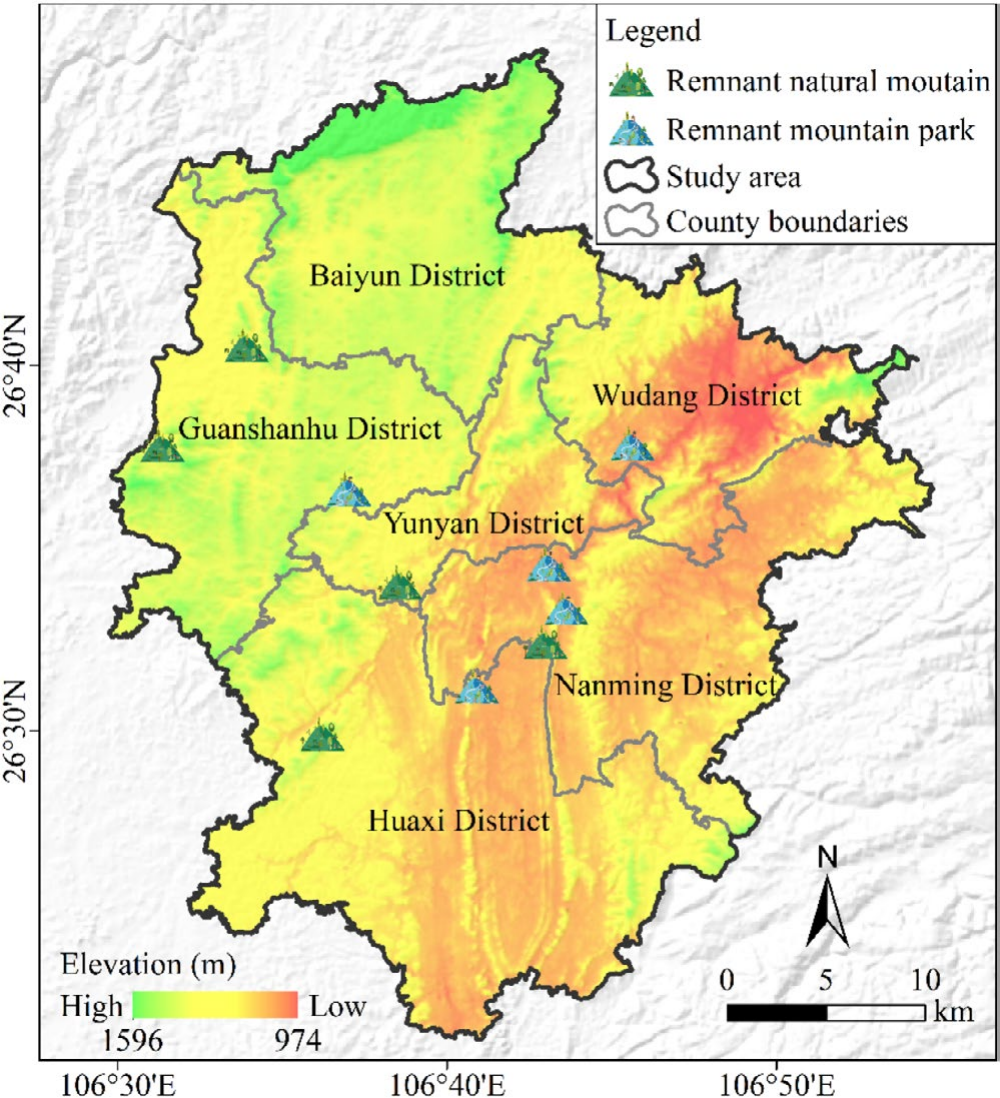
Location of the study area.

Guiyang City is situated in the central part of Guizhou Province, China, and is a representative karst mountainous city in the southwest of China (Liu et al. 2022a; Wang et al. 2022b). The central urban area (26°21′–26°48′ N, 106°30′–106°55′ E) encompasses an area of approximately 1180 km^2^ (Wang et al. 2022a) (Figure 1). The climate zone is classified as a subtropical monsoon, with an average annual temperature of approximately 15.3°C and an average annual precipitation ranging from 1197 to 1248 mm (Meng et al. 2016). Mountainous regions account for approximately 52.30% of the total area, with an average elevation of around 1200 m (Luo et al. 2022; Wang et al. 2022a). The predominant soil types are yellow earth and calcareous soil, characterized by shallow soil layers with limited water retention and storage capacity (Pan et al. 2017). By 2020, Guiyang City had achieved a vegetation coverage of 50%, featuring diverse vegetation types and rich biodiversity, which includes a total of 2307 families, 979 genera, and 2908 species of vascular plants (Wang et al. 2022b). The vegetation types are primarily categorized into coniferous forests, broadleaved forests, and shrubs (or shrub‐grasslands), with the zonal vegetation being subtropical evergreen broadleaved forests. Between 2010 and 2020, the population surged by 38.35%, coinciding with a rapid pace of urbanization, during which the urban area expanded by 45.26% (Wu et al. 2024). In recent years, with the accelerating urbanization and the vigorous promotion of the development and utilization of mountain parks by the Guiyang Municipal Government, a large number of karst mountains have been forced into urban areas, while the remaining urban mountains have been subject to fragmentation, degradation, and erosion (Tang and Wang 2021; Wang et al. 2022a; Zeng et al. 2022).

### 2.2 Plant Communities Investigation

This study utilized Gaofen‐1 satellite remote sensing imagery (2 m × 2 m resolution, 2020) to construct a spatial attribute database of urban remnant mountains in Guiyang, using ArcGIS 10.5 for visual interpretation and field validation. Gaofen‐1 was selected due to its high spatial resolution and extensive coverage, which effectively supports urban remnant mountain identification and spatial database construction. By referencing relevant studies (Zeng et al. 2022) and considering the specific conditions of the study area, we selected five URNM and five URMP from the spatial attribute database. The selection criteria ensured consistency in the degree of surrounding impervious surfaces, forest type (natural secondary forest), mountain area, and soil type.

To examine the plant diversity of typical remnant mountains, a systematic sampling method was employed, utilizing transect and site survey techniques (Tang and Wang 2021; Zeng et al. 2022). Transects were established in both horizontal and vertical orientations to ensure representativeness and comprehensiveness of the samples. Horizontally, “+”‐shaped survey transects extended from the edge to the center of each mountain along the four cardinal directions: east, west, north, and south. Vertically, a transect was established along slope positions (upper slope (US), middle slope (MS), and lower slope (LS)) from the US to the LS of each mountain. This design ensured a comprehensive representation of plant diversity across spatial gradients. Each sample plot measured 30 m × 30 m, with nine such plots designated within each remnant mountain, resulting in a total of 90 sample plots. Within each plot, three or four nested quadrats were arranged at intervals of no less than 3 to 5 m. The quadrat sizes were as follows: 10 m × 10 m for the tree layer, 5 m × 5 m for the shrub layer, and 2 m × 2 m for the herb layer. All plants within the quadrats, encompassing the tree, shrub, and herb layers, were surveyed. In the tree layer (diameter at breast height (DBH) ≥ 5 cm), species names, abundance, heights (m), and DBH (m) were recorded (Tang and Wang 2021). For the shrub layer (including small trees with DBH < 5 cm), species names, abundance, average heights (m), and coverage (%) were measured and recorded. The herb layer survey included species names, abundance, average heights (m), and coverage (%). Species abundance data were used to calculate and analyze plant diversity, while other data were used to calculate and analyze the importance value indices of species. Additionally, the longitude, latitude, elevation, and slope information of each quadrat were recorded using GPS. Considering the abundance of cultivated plants within the mountain parks, plant assemblages were categorized into three distinct types: all plants present in the urban remnant mountain parks (URMPA), wild plants occurring naturally within the urban remnant mountain parks (URMPW), and cultivated plants within the urban remnant mountain parks (URMPC).

### 2.3 Soil Samples Collection and Determination

Soil samples from a depth of 0–20 cm were collected using a 100 cm^3^ diameter ring knife, following a five‐point mixed sampling approach in each plot. Six samples were collected at each location, with three dedicated to determining soil bulk density (SBD). The remaining three samples were transported to the laboratory for the analysis of additional soil parameters. To eliminate the influence of rainfall on soil moisture content (SMC) and SBD, we ensured that no precipitation occurred during the week preceding the sampling day.

SMC was measured using the drying method; SBD was measured via the cutting ring method; soil organic carbon (SOC) was determined by the potassium dichromate hydration heating method; soil total nitrogen (TN) was analyzed using the Kjeldahl method; soil total phosphorus (TP) was measured using an acid digestion method with H_2_SO_4_ and HClO_4_ solutions; and soil total potassium (TK) was determined using flame photometry with NaOH melting (Xiao et al. 2023; Yaseen et al. 2022). In addition, we calculated the soil C/N, C/P, and N/P ratios.

### 2.4 Plant Diversity Indexes Calculation

This study employs the importance value indices (*IVI*) to evaluate the species composition and structure of the community (Ma et al. 1995). The calculation formulas for each index are as follows:

(1) $${\mathrm{RD}}_{i}=D_{i}/\sum D$$

(2) $${\mathrm{RH}}_{i}=H_{i}/\sum H$$

(3) $$\mathrm{RD}m_{i}={\mathrm{BA}}_{i}/\sum \mathrm{BA}$$

(4) $${\mathrm{RC}}_{i}=C_{i}/\sum C$$

(5) $${\mathrm{IVI}}_{i}\left(\text{trees}\right)=\left({\mathrm{RD}}_{i}+{\mathrm{RH}}_{i}+{\mathrm{RDm}}_{i}\right)/3$$

(6) $${\mathrm{IVI}}_{i}\left(\text{shrubs and herbs}\right)=\left({\mathrm{RH}}_{i}+{\mathrm{RC}}_{i}\right)/2$$

where *RD*_*i*_ is the relative density of species *i*, *D*_*i*_ is the density of species *i*, ∑*D* is the sum of the densities of all species, *RH*_*i*_ is the relative height of species *i*, *H*_*i*_ is the average height of species *i*, ∑*H* is the sum of the average heights of all species, *RDm*_*i*_ is the relative dominance of species *i*, *BA*_*i*_ is the basal area at breast height of species *i*, ∑*BA* is the sum of the basal areas at breast height of all species, *RC*_*i*_ is the relative cover of species *i*, *C*_*i*_ is the average cover of species *i*, ∑*C* is the sum of the average covers of all species, and *IVI*_*i*_ is the importance value for the *i*th species.

Additionally, the Margalef index (*R*), Shannon–Wiener index (*H*), Simpson index (*D*), and Pielou index (*E*) are employed to calculate plant diversity (Ma et al. 1995; Yaseen et al. 2022). The calculation formulas for each index are as follows:

(7) $$R=\frac{\left(S-1\right)}{\ln N}$$

(8) $$H=-\sum_{i=1}^{S}P_{i}\ln P_{i}$$

(9) $$D=1-\sum_{i=1}^{S}P_{i}^{2}$$

(10) E=H/lnS

(11) $$P_{i}=N_{i}/N$$

where *S* is the species number, *N*_*i*_ is the individual number of the *i*th species, and *N* is the individual number of all species.

### 2.5 Statistical Analysis

Nonparametric Kruskal–Wallis analyses were systematically conducted to assess plant diversity gradients and edaphic heterogeneity across three types of urban karst remnant mountains (URNM, URMPA, URMPW), given the violation of parametric assumptions (Shapiro–Wilk test, *p* < 0.05). To investigate the relationships between plant diversity and soil environmental factor characteristics, Spearman's correlation analysis was conducted. Moreover, redundancy analysis (RDA) was performed using Canoco 5.0 software to assess the impact of soil environmental factors on plant diversity among the different types of urban remnant mountains. Data analysis and visualization were conducted using Origin 2021 and R 4.3.3, respectively.

## 3 Results

### 3.1 Plant Species Composition of Urban Remnant Mountains

**FIGURE 2:**
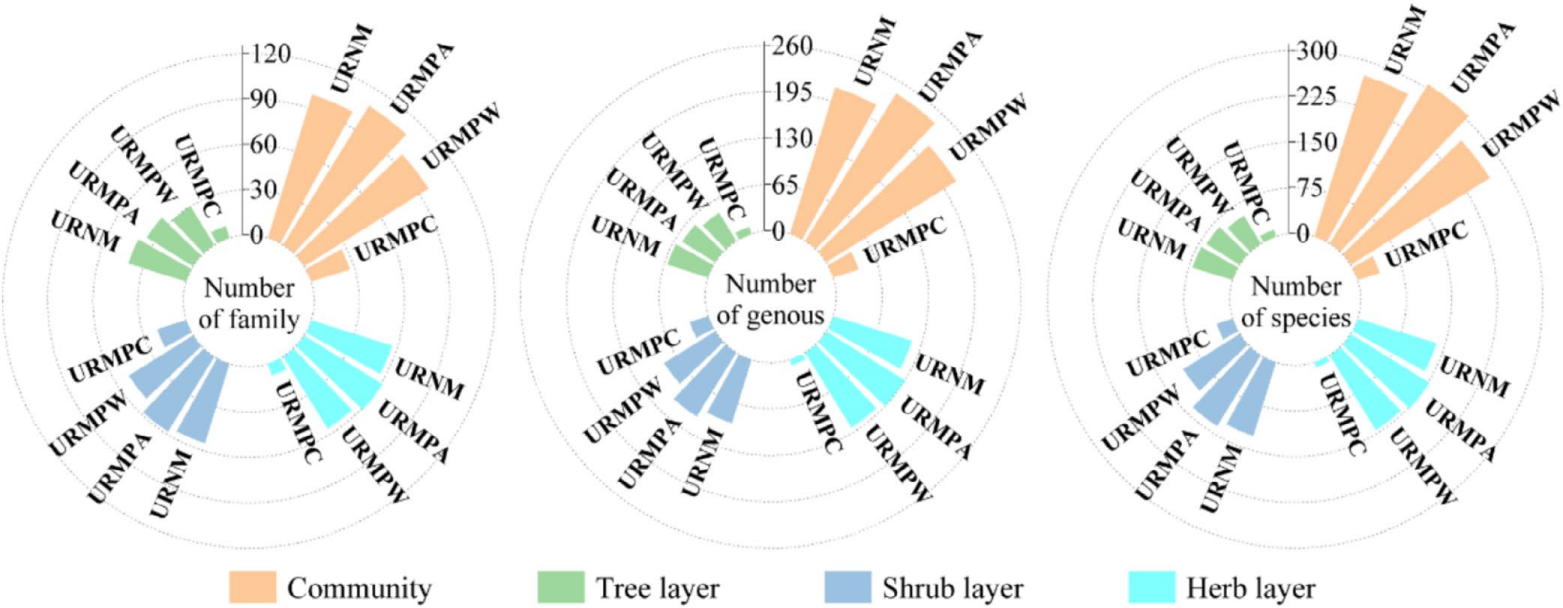
Species composition of plant communities in different types of urban remnant mountains. URMPA, all plants present in the urban remnant mountain parks; URMPC, cultivated plants within the urban remnant mountain parks; URMPW, wild plants occurring naturally within the urban remnant mountain parks; URNM, urban remnant natural mountains.

**FIGURE 3:**
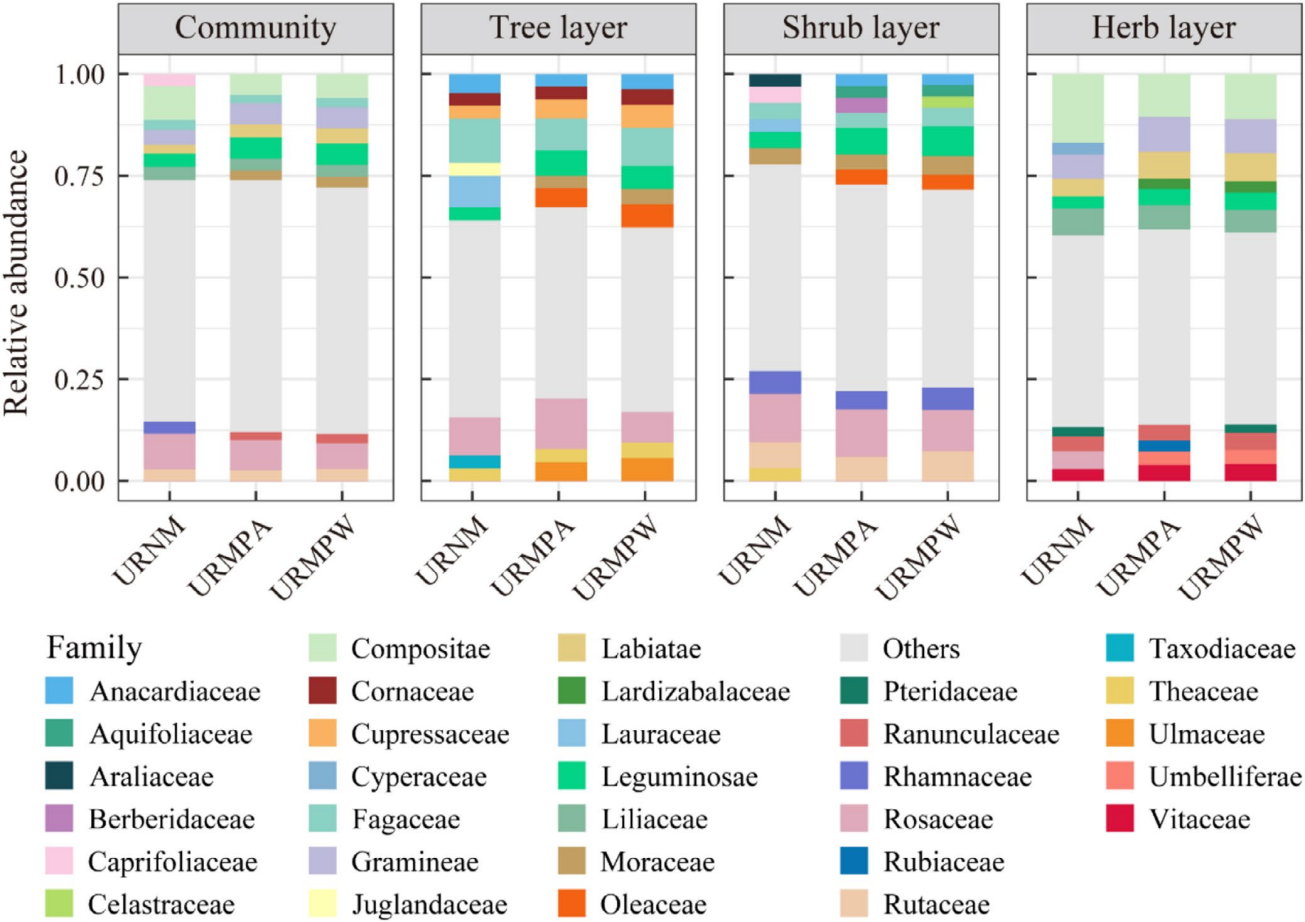
Main families in plant communities of different types of urban remnant mountains. URMPA, all plants present in the urban remnant mountain parks; URMPW, wild plants occurring naturally within the urban remnant mountain parks; URNM, urban remnant natural mountains.

**FIGURE 4:**
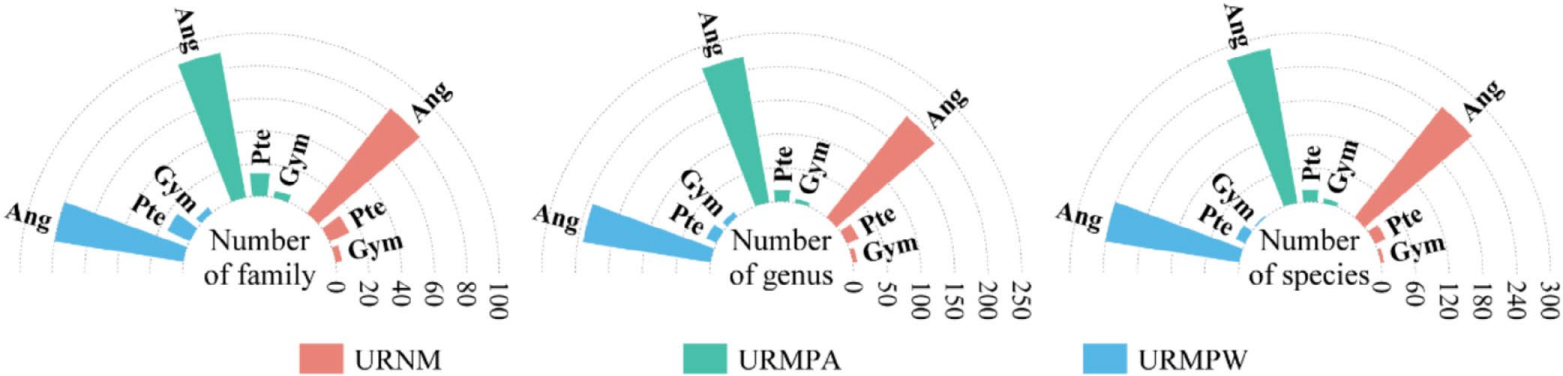
Vegetation groups in different types of urban remnant mountains. Ang, Angiosperms; Gym, Gymnospermia; Pte, Pteridophyte; URMPA, all plants present in the urban remnant mountain parks; URMPW, wild plants occurring naturally within the urban remnant mountain parks; URNM, urban remnant natural mountains.

A total of 276 vascular plant species were recorded across 99 families and 215 genera in the five URNM (Figure 2). By comparison, the URMPA hosted 307 species from 108 families and 243 genera, while the URMPW contained 269 species across 97 families and 215 genera. Rosaceae, Compositae, and Gramineae were the most abundant families in all remnant mountains (Figure 3), with angiosperms constituting over 87.32% of the total species in each category (Figure 4). Overall, species diversity was slightly higher in URMPA, with the highest abundance of angiosperms across all remnant mountains.

The composition of life forms varied across the different types of remnant mountains. The tree layer in URNM was dominated by Fagaceae, Rosaceae, and Lauraceae (Figure 3), while Fagaceae, Rosaceae, and Cupressaceae were the dominant families in the tree layers of both URMPA and URMPW. The shrub layer in URNM featured Rosaceae, Rutaceae, and Rhamnaceae, while the shrub layers in URMPA and URMPW were led by Rosaceae, Leguminosae, and Rutaceae. In terms of the herb layer, URNM was mainly dominated by Compositae, Liliaceae, and Gramineae, whereas Compositae, Gramineae, and Labiatae prevailed in URMPA and URMPW. These results highlight the variability in plant composition across layers and between different types of remnant mountains, with certain families being dominant in specific plant layers.

**FIGURE 5:**
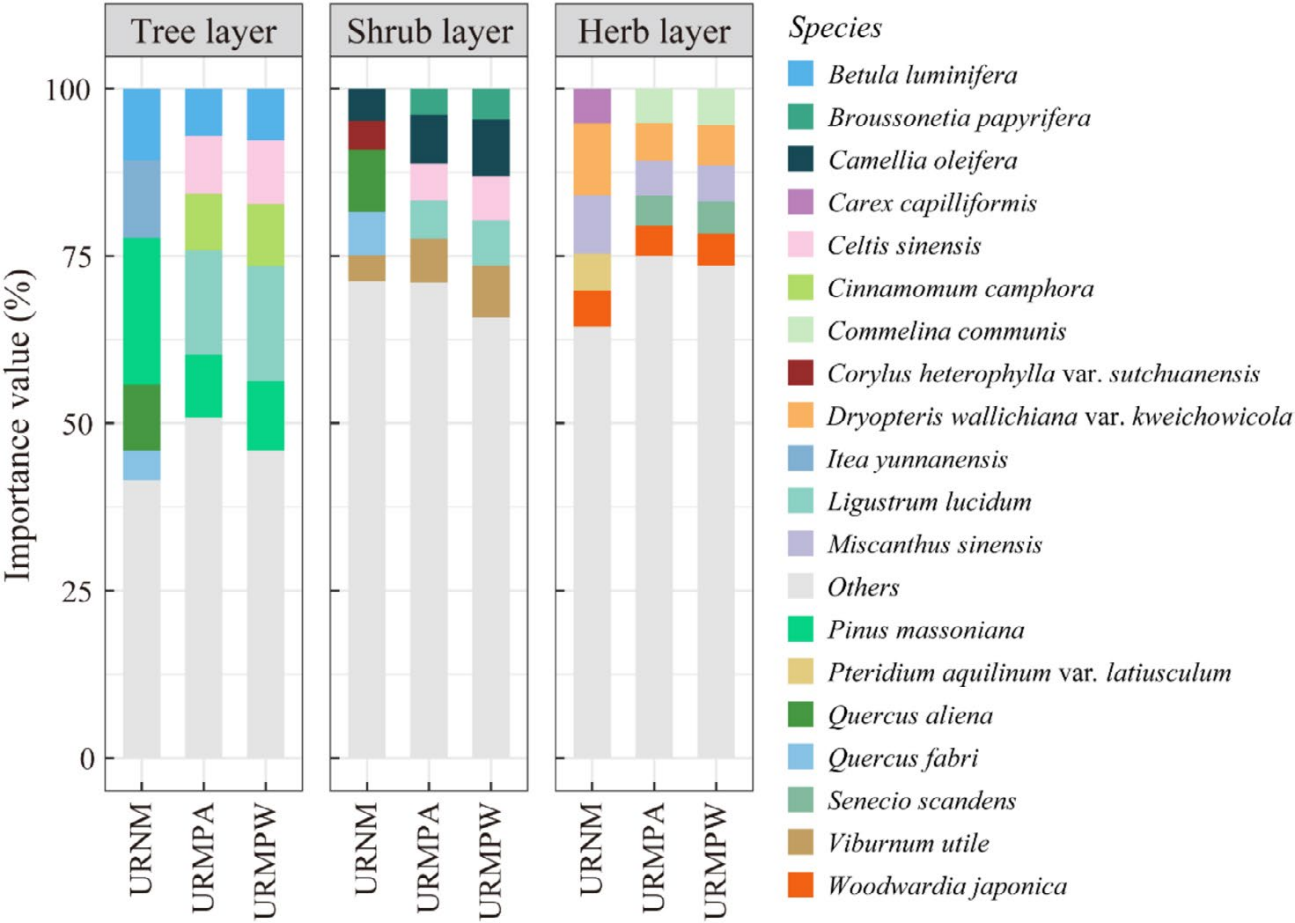
Dominant plant species and their importance values in different types of urban remnant mountains. URMPA, all plants present in the urban remnant mountain parks; URMPW, wild plants occurring naturally within the urban remnant mountain parks; URNM, urban remnant natural mountains.

In the tree layer of URNM, 69 species from 60 genera and 40 families were recorded, with *Pinus massoniana* , *Itea yunnanensis*, and *Betula luminifera* as dominant species (Figure 2 and Figure 5). The shrub layer was more diverse, comprising 126 species from 93 genera and 55 families, including *Quercus aliena*, *Quercus fabri*, and *Camellia oleifera*. The herb layer, being the most species‐rich, included 136 species from 116 genera and 56 families, with *Dryopteris wallichiana* var. *kweichowicola*, *Miscanthus sinensis* , and *Pteridium aquilinum var. latiusculum* as the leading species. These layers represented 25.00%, 45.65%, and 49.28% of the total species in URNM, respectively. In contrast, the tree layer of URMPA was less species‐diverse, with 64 species from 11 genera and 8 families, dominated by *Ligustrum lucidum* , *P. massoniana* , and *Celtis sinensis* (Figure 5). The shrub layer in URMPA was notably more diverse, comprising 136 species from 105 genera and 58 families, with species like *C. oleifera* , *Viburnum utile*, and *L. lucidum* . The herb layer in URMPA, which was the most species‐rich, included 152 species from 130 genera and 61 families, with *D. wallichiana* var. *kweichowicola*, *Commelina communis* , and *M. sinensis* as the leading species. These life forms represented 20.85%, 44.30%, and 49.51% of the total species richness in URMPA, respectively. The analysis reveals that the herb layer consistently showed the highest species richness, followed by the shrub layer, while the tree layer exhibited the lowest diversity across both URNM and URMPA.

The shrub and herb layers of URMPA exhibited significantly higher species richness compared to URNM. A total of 269 wild plant species were identified in URMPA, representing 87.62% of the species pool, with 53 species in the tree layer, 109 in the shrub layer, and 144 in the herb layer. Additionally, 38 artificially planted species were recorded, making up 12.38% of the total species count, with 11 species in the tree layer, 27 in the shrub layer, and 8 in the herb layer. This highlights the significantly greater diversity in the shrub and herb layers of URMPA, with a notable proportion of artificially planted species contributing to the overall richness.

### 3.2 Distribution Pattern of Plant Diversity in Urban Remnant Mountains

**FIGURE 6:**
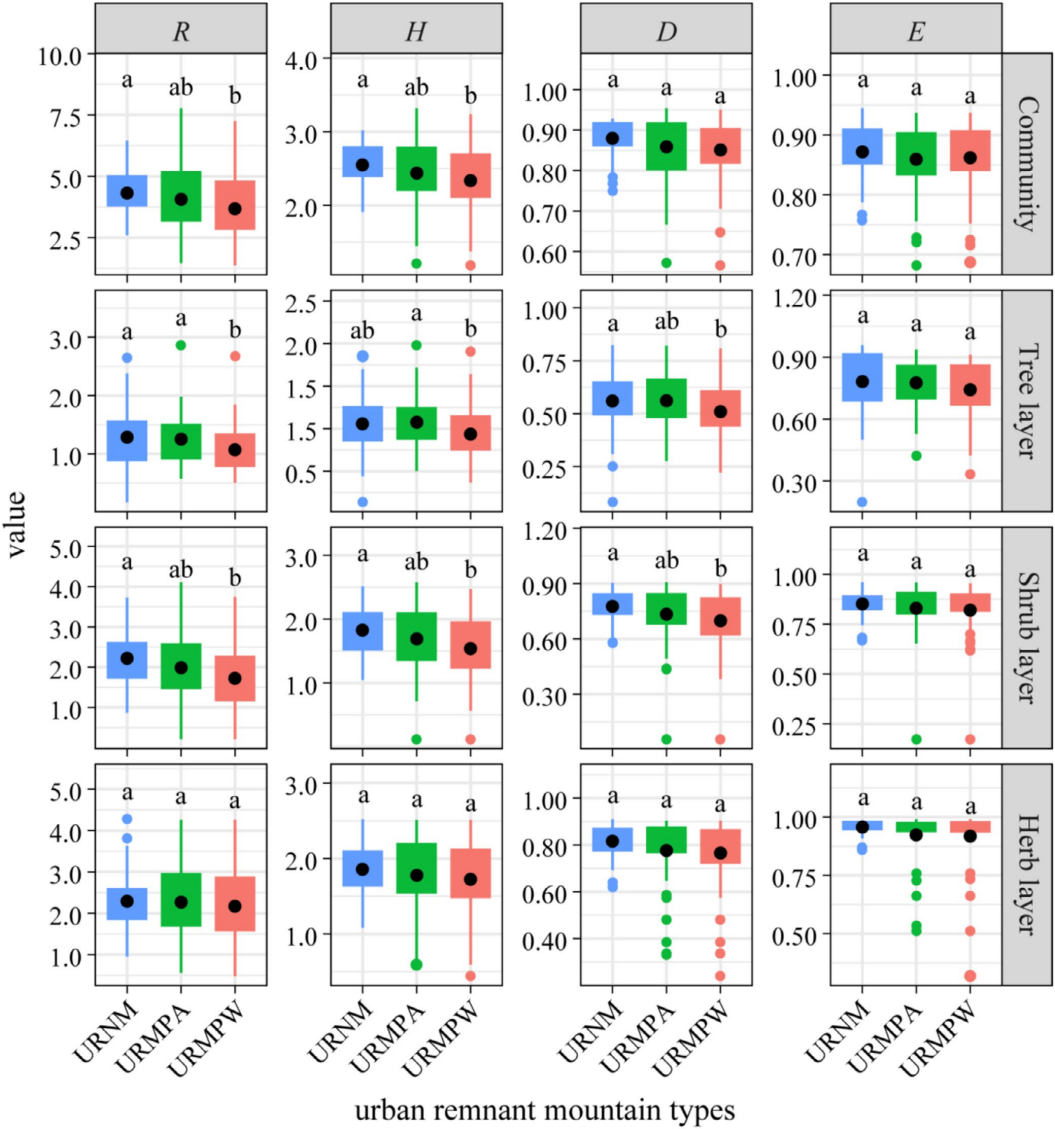
Plant diversity of different life forms in different urban remnant mountains. *D*, Simpson index; *E*, Pielou index; *H*, Shannon‐Wiener index; *R*, Margalef index; URMPA, all plants present in the urban remnant mountain parks; URMPW, wild plants occurring naturally within the urban remnant mountain parks; URNM, urban remnant natural mountains. Different lowercase letters indicate a significant difference (*p* < 0.05).

The plant diversity across different types of remnant mountains revealed a distinctive pattern, with URNM exhibiting greater diversity than both URMPA and URMPW (Figure 6). The *R* and *H* of the URNM community, as well as the *R*, *H*, and *D* indices in the tree and shrub layers, were significantly higher compared to those in URMPW (*p* < 0.05). This indicates that, compared to URMPA and URMPW, URNM generally supports higher plant diversity, particularly in the tree and shrub layers.

**FIGURE 7:**
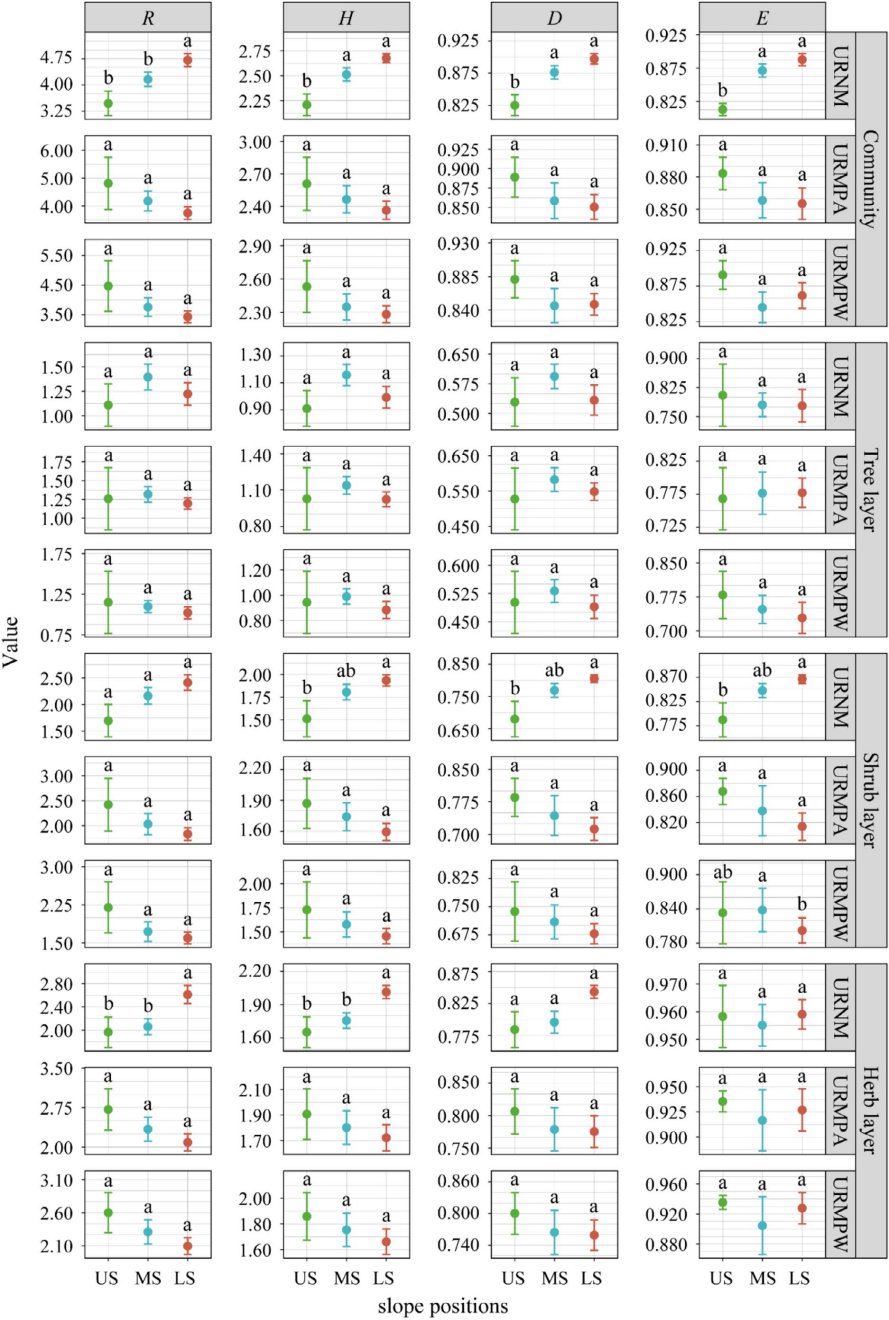
Plant diversity at different slope positions in various types of urban remnant mountains. *D*, Simpson index; *E*, Pielou index; *H*, Shannon–Wiener index; LS, lower slope; MS, middle slope; *R*, Margalef index; URMPA, all plants present in the urban remnant mountain parks; URMPW, wild plants occurring naturally within the urban remnant mountain parks; URNM, urban remnant natural mountains; US, upper slope. Different lowercase letters indicate significant difference (*p* < 0.05).

In terms of vertical distribution, plant diversity in the URNM community, shrub layer, and herb layer increased from the US to the LS (Figure 7). In contrast, the diversity distribution in URMPA and URMPW followed an opposite trend, with diversity decreasing from US to LS. Interestingly, the tree layer's plant diversity distribution in both URMPA and URMPW showed patterns similar to those observed in URNM.

### 3.3 Distribution Pattern of Soil Environmental in Urban Remnant Mountains

**FIGURE 8:**
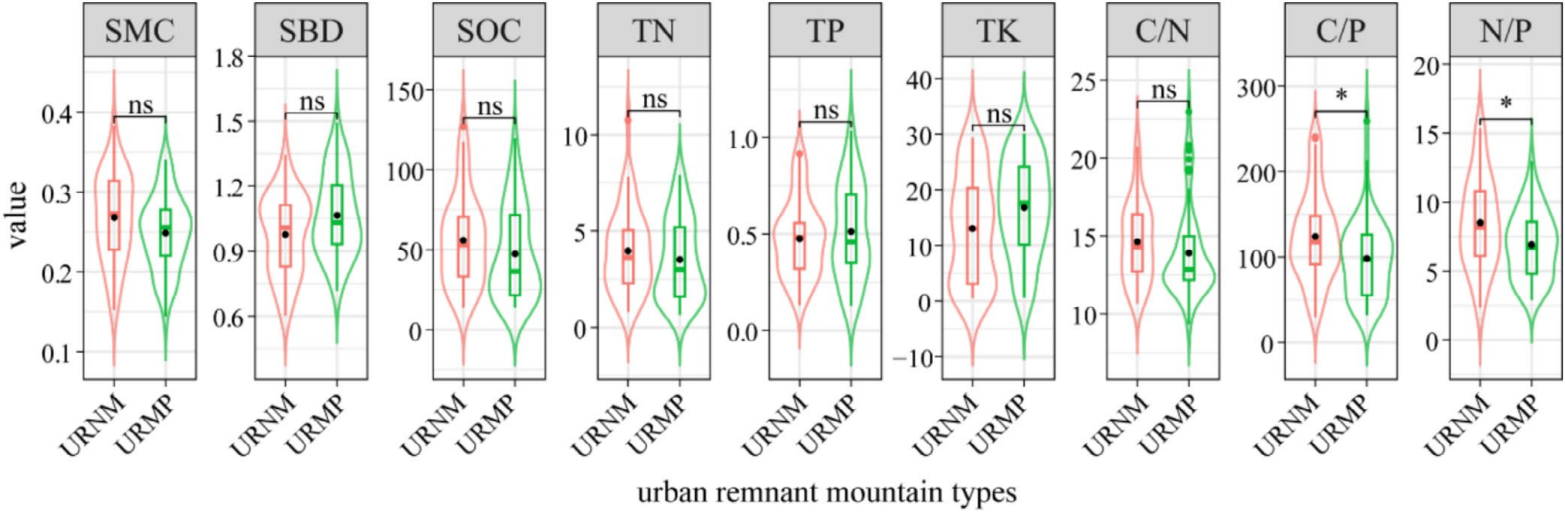
Soil environmental factors in different urban remnant mountains. C/N, Carbon to nitrogen ratio; C/P, Carbon to phosphorus ratio; N/P, Nitrogen to phosphorus ratio; SBD, Soil bulk density (g/cm^3^); SMC, Soil moisture content (%); SOC, Soil carbon content (g/kg); TK, Total potassium (g/kg); TN, Total nitrogen (g/kg); TP, Total phosphorus (g/kg); URMP, Urban remnant mountain packs; URNM, Urban remnant natural mountains. *p < 0.05; ns, *p* > 0.05.

The SMC, SOC, and TN contents of URNM were slightly higher than those of URMP, while the contents of SBD, TP, and TK were slightly lower (Figure 8). Moreover, the stoichiometric ratios (C/N, C/P, and N/P) of URNM were all higher than those of URMP, with the C/P and N/P ratios being significantly higher (*p* < 0.05). This indicates that, compared to URMP, URNM has higher carbon and nitrogen contents, as well as more favorable stoichiometric ratios, which could potentially influence nutrient cycling and ecosystem functions.

### 3.4 Relationship Between Plant Diversity and Soil Environment in Urban Remnant Mountains

**FIGURE 9:**
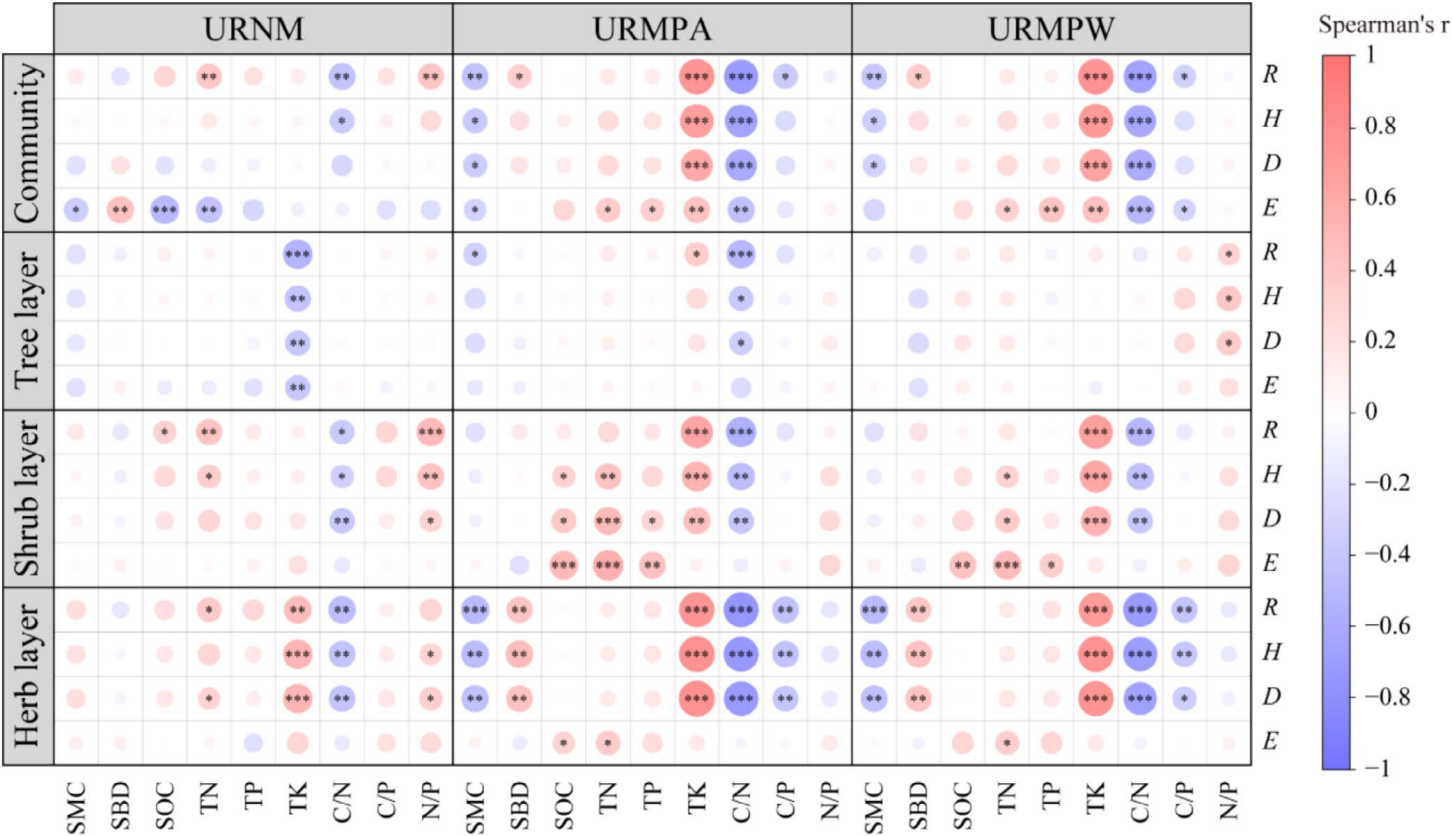
Correlations between plant diversity of different life forms and soil environmental factors in various types of urban remnant mountains. C/N, Carbon to nitrogen ratio; C/P, Carbon to phosphorus ratio; D, Simpson index; E, Pielou index; H, Shannon‐Wiener index; N/P, Nitrogen to phosphorus ratio; R, Margalef index; SBD, Soil bulk density; SMC, soil moisture content; SOC, soil carbon content; TK, total potassium; TN, total nitrogen; TP, total phosphorus; URMPA, all plants present in the urban remnant mountain parks; URMPW, wild plants occurring naturally within the urban remnant mountain parks; URNM, urban remnant natural mountains. *p < 0.05, **p < 0.01, ***p < 0.001.

The correlation between plant diversity indices and soil environmental characteristics varied significantly across different types of urban remnant mountains (Figure 9). There were only eight significant correlations (*p* < 0.05) between the soil environment and the plant diversity in URNM communities, while both URMPA and URMPW had as many as 16 significant correlations (*p* < 0.05) with the soil environment. In addition, the number of significant correlations (*p* < 0.05) between soil environmental factors and plant diversity in URMN, URMPA, and URMPW showed a characteristic of the herb layer being greater than the shrub layer and the tree layer.

**FIGURE 10:**
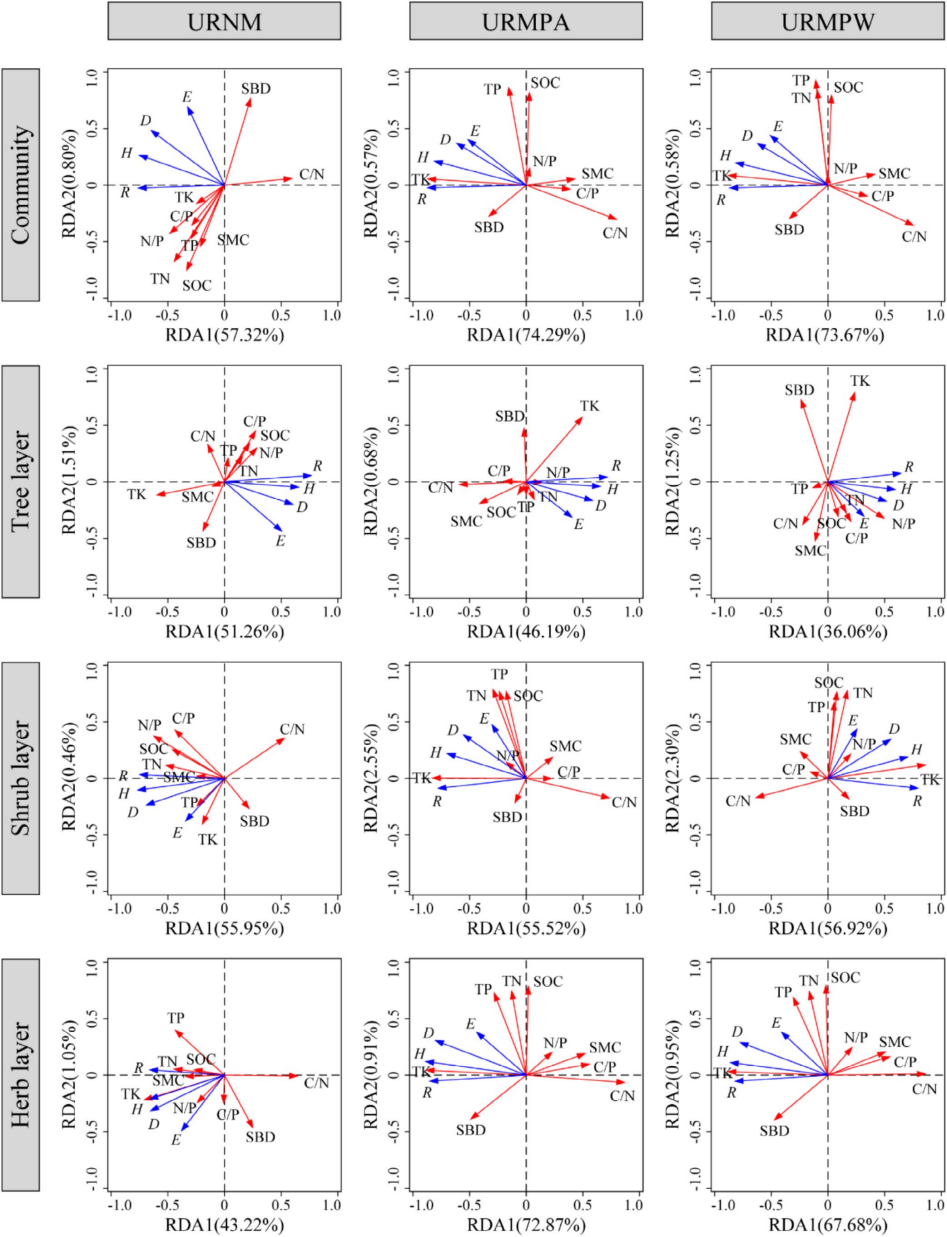
RDA ordination of plant diversity in different life forms and soil environmental factors in various types of urban remnant mountains. C/N, Carbon to nitrogen ratio; C/P, Carbon to phosphorus ratio; D, Simpson index; E, Pielou index; H, Shannon‐Wiener; N/P, Nitrogen to phosphorus ratio; R, Margalef index; SBD, soil bulk density; SMC, soil moisture content; SOC, soil carbon content; TK, total potassium; TN, total nitrogen; TP, total phosphorus; URMPA, all plants present in the urban remnant mountain parks; URMPW, wild plants occurring naturally within the urban remnant mountain parks; URNM, urban remnant natural mountains.

**FIGURE 11:**
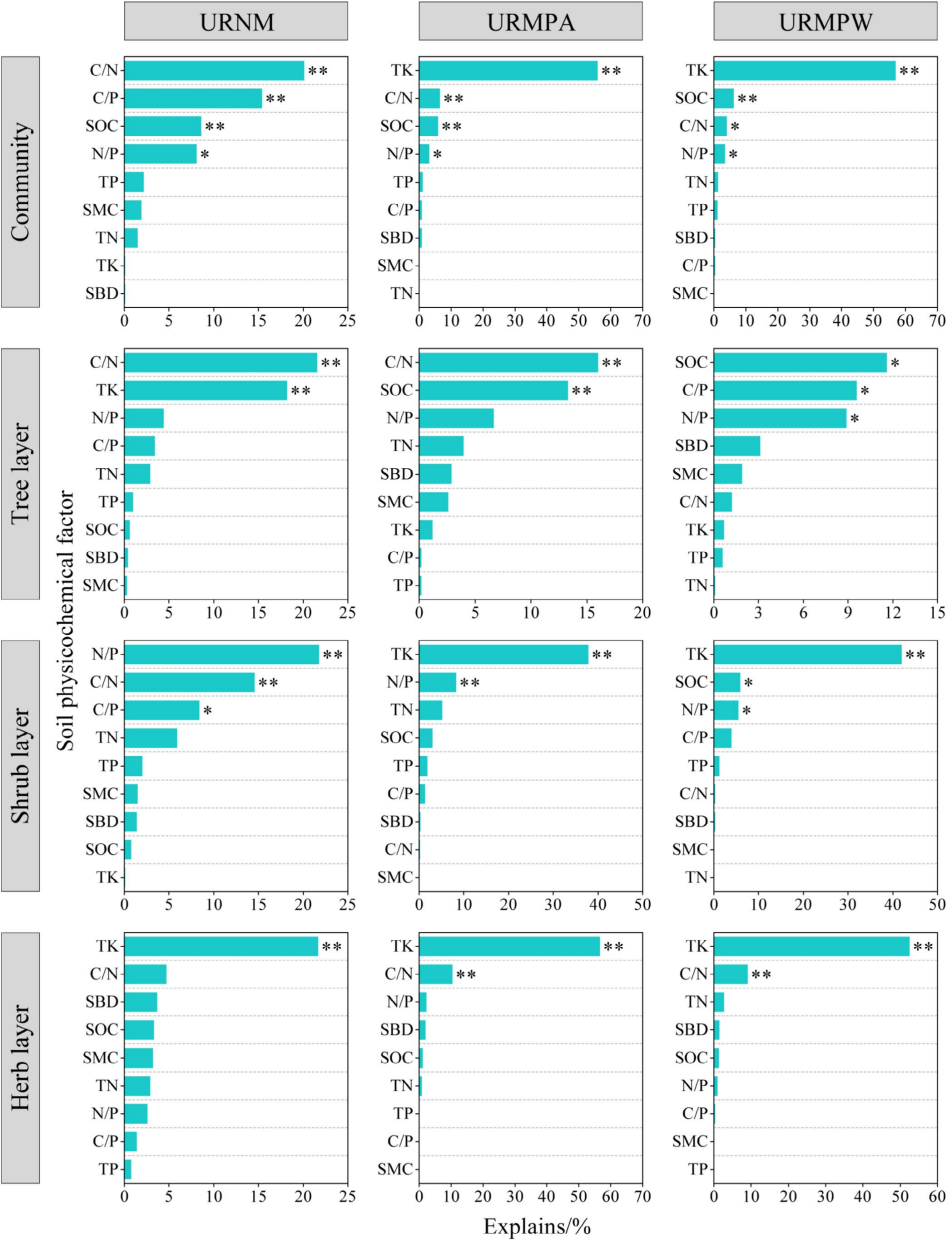
Explanatory power of soil environmental factors on plant diversity in different life forms across various types of urban remnant mountains. C/N, Carbon to nitrogen ratio; C/P, Carbon to phosphorus ratio; D, Simpson index; E, Pielou index; H, Shannon‐Wiener index; N/P, Nitrogen to phosphorus ratio; R, Margalef index; SBD, soil bulk density; SMC, soil moisture content; SOC, soil carbon content; TK, total potassium; TN, total nitrogen, TP, total phosphorus; URMPA, all plants present in the urban remnant mountain parks; URMPW, wild plants occurring naturally within the urban remnant mountain parks; URNM, urban remnant natural mountains. *p < 0.05, **p < 0.01.

The RDA analysis revealed significant variations in soil environmental factors influencing plant diversity across different life forms among urban remnant mountains (Figure 10). For URNM, URMPA, and URMPW communities, the cumulative explanatory power of the first two RDA axes reached 58.12%, 74.86%, and 74.25%, respectively. Notably, soil factors in URNM exhibited the strongest explanatory capacity for shrub layer diversity (56.41%) yet the weakest for herb layer diversity (44.27%). Conversely, URMPA and URMPW demonstrated contrasting patterns, with soil factors explaining the highest variance in herb layer diversity (73.78% and 68.63%, respectively) and the lowest in tree layer diversity (46.87% and 37.31%, respectively). Detailed analysis identified TK as the dominant driver influencing herb layer diversity in URNM, along with shrub and herb layer diversity in URMPA and URMPW, contributing 21.7%–56.9% independent explanatory power (*p* < 0.01) (Figure 11). The tree layer diversity in URMPA was primarily governed by C/N ratio and SOC, accounting for 16.0% and 13.3% variance, respectively (*p* < 0.01). In URMPW, tree layer diversity showed significant associations with SOC, C/P, and N/P ratios, each explaining > 8.9% variation (*p* < 0.05). For overall plant diversity in URNM, C/N ratio (20.1%), C/P ratio (15.4%), and SOC (8.6%) emerged as key determinants (*p* < 0.01). Shrub layer diversity in URNM was jointly influenced by N/P ratio (21.8%), C/N ratio (14.6%), and C/P ratio (8.4%) (*p* < 0.05), while tree layer diversity predominantly responded to C/N ratio (21.6%) and TK (18.2%) (*p* < 0.01).

## 4 Discussion

Our research findings indicate that the transformation of karst urban natural remnant mountains into mountain parks leads to a simplification of plant species composition, a reduction in plant diversity, and changes in the spatial patterns of plant diversity. Moreover, the main soil physicochemical factors influencing plant community diversity in URNM ecosystems, including C/N, C/P, and SOC, differ from those influencing plant diversity in URMP, such as TK. Human disturbances were found to amplify the correlation between soil properties and plant diversity.

### 4.1 Distribution Pattern of Plant Diversity in Urban Remnant Mountains

Human disturbance intensity represents a key determinant of ecosystem dynamics, critically shaping community structure and driving shifts in species diversity (Cardinale et al. 2012; Isbell et al. 2017; Western 2001). Our analysis demonstrated substantial variation in vascular plant composition across remnant mountain types: URMPA contained 307 species, exceeding URNM (276) and URMPW (269) (Figure 2). Notably, URMPA's apparent richness advantage disappeared when cultivated plants were excluded, yielding 269 wild species, a count closely aligned with the 265 species reported in Maolan karst forests (Li et al. 2022). Taxonomic dominance patterns exhibited spatial differentiation, with Fagaceae, Rosaceae, and Lauraceae emerging as the most species‐rich families in URNM, URMPA, and URMPW, respectively (Figure 3), a distribution pattern consistent with Japanese forest studies (Hayasaka et al. 2012). These dominant families display adaptive strategies combining disturbance tolerance and r‐selected reproductive traits (Chen et al. 2014; Li and Chen 2002), facilitating their proliferation in heterogeneous environments. Plant life forms across remnant mountain types showed distinct patterns: herbs and shrubs dominated, while trees were least abundant (Figure 2). This lower tree diversity likely stems from their stricter habitat needs, contrasting with shrubs and herbs that thrive due to shorter lifespans, faster regeneration, and adaptability to limited space (Körner 2012; Tian et al. 2015).

The observed reduction in plant diversity in mountain park supports findings that human disturbance can reduce plant diversity (Wan et al. 2024; Yang et al. 2021). This study similarly showed that plant diversity in different types of remnant mountains followed the characteristic of URNM being greater than URMPA and URMPW, with URNM exhibiting a richer plant composition than URMPW (Figures 3 and 6). The conversion of remnant mountains into parklands leads to the destruction of native vegetation by park facilities (e.g., roads, buildings), which reduces native species, alters community structure, and ultimately lowers plant diversity (Cheng et al. 2022; LaPaix and Freedman 2010). Moreover, human management practices in parks, such as pruning, watering, and fertilizing, may enhance the diversity of cultivated plants but suppress the diversity of wild plants, further contributing to a decrease in overall plant diversity (Guo et al. 2024; Wheeler et al. 2017).

Slope position plays a significant role in shaping the distribution patterns and diversity of plants by regulating various environmental factors (Feng et al. 2018; Sariyildiz et al. 2005). In URNM, LS exhibited greater diversity than MS, which in turn had greater diversity than US (Figure 7). This pattern aligns with studies from the central Himalayan forests (Tyagi et al. 2023), where LS areas typically have more stable microclimates and richer ecological niches, supporting a greater variety of plant species (Shovon et al. 2020; Wang et al. 2023). As slope position increases, environmental conditions become harsher, which limits plant growth and reduces diversity (Liu et al. 2023). Interestingly, in URMPA and URMPW, the highest plant diversity was found at US (Figure 6), primarily due to significant human disturbances at the LS, where many native species were replaced by ornamental plants (Htun et al. 2011; Liu et al. 2023; Muratet et al. 2015).

### 4.2 Distribution Pattern of Soil Environmental Factors in Urban Remnant Mountains

Soil plays a fundamental role in maintaining plant diversity and ecosystem functions (Smith et al. 2015). Our study found that URMP exhibited significantly higher SBD, TP, and TK compared to URNM (Figure 8), likely due to compaction from human activities, such as tourism, fertilization, and infrastructure development (Cheng et al. 2022; Kissling et al. 2009; Sujetovienė and Baranauskienė 2016).

On the other hand, URNM, with minimal human disturbance, retains a more natural soil structure, exhibiting lower SBD and relatively lower levels of TP and TK. The absence of artificial nutrient additions allows for a more balanced nutrient cycle, where plant decomposition contributes to soil organic matter, enriching the soil naturally. In URNM, the dense vegetation cover reduces soil erosion, SMC, and facilitates the accumulation of organic matter. This organic matter not only improves soil structure but also releases essential nutrients, such as carbon and nitrogen, during decomposition, thereby increasing SOC and TN levels (Hu et al. 2018; Saint‐Laurent et al. 2014; Zhou et al. 2019). These processes, driven by low human interference, promote a healthy microbial community and a dynamic nutrient cycling system. The higher C/N, C/P, and N/P ratios observed in URNM reflect a more stable and natural nutrient cycle, supporting greater plant diversity. These higher ratios suggest that URNM's soil is richer in organic material, where nutrients are stored in a more balanced form, thus enhancing nutrient availability for plants (Chen and Chen 2021; Dang et al. 2023; Navarrete et al. 2023).

In contrast, human management activities in URMP, such as frequent pruning, fertilization, and vegetation management, disturbed the natural nutrient cycling processes. Over‐fertilization, in particular, could have led to an imbalance in soil nutrient ratios, particularly reducing C/P and N/P ratios. These imbalances indicated that nutrients like phosphorus and nitrogen were no longer available in the optimal proportions for supporting a diverse plant community (Heyburn et al. 2017; Huang et al. 2021; Kim et al. 2023).

### 4.3 Relationship Between Plant Diversity and Soil Environmental Factors in Urban Remnant Mountains

This study found that human disturbance has a profound impact on the relationship between soil properties and plant diversity in karst urban remnant mountains and transformed mountain parks. Specifically, compared to the natural state of URNM, the correlation between soil environmental factors and plant diversity was stronger in URMP, with both URMPA and URMPW showing 16 significant correlations between soil factors and plant diversity, compared to only eight in URNM (Figure 9). Moreover, the soil environmental factors explained more than 74% of the variation in plant diversity in the transformed parks, compared to 58.12% in URNM (Figure 10). These findings indicate that compared to the natural state, human disturbance has strengthened the correlation between soil environmental factors and plant diversity, which is consistent with the findings of Bisht et al. (2022).

The enhanced coupling between soil factors and plant diversity in disturbed areas is primarily driven by the changes in soil structure and nutrient cycling caused by human activities such as construction, tourism, fertilization, and vegetation management (Li and Wang 2016; Wang et al. 2024; Wangchuk et al. 2014). These activities directly alter the physical and chemical properties of the soil, which in turn affect the availability of nutrients. The increased significance of soil properties in URMP likely reflects the greater dependency of plant communities on altered soil conditions, as human interventions such as soil compaction, fertilization, and vegetation removal disrupt the natural buffering mechanisms that typically stabilize soil environments. Additionally, the herbaceous and shrub layers in URMP showed stronger correlations with soil factors than the tree layer. This difference can be attributed to the higher sensitivity of smaller, faster‐growing plants to soil changes. Herbs and shrubs, with their shorter life cycles and lower resilience to disturbance, are more directly affected by fluctuations in soil nutrients and structure (Xing et al. 2022). Trees, on the other hand, tend to have deeper root systems and longer life spans, making them more resistant to immediate soil changes. Our analyses identified stoichiometric ratios (C/N, C/P, N/P) and SOC as primary determinants of plant diversity in URNM communities (Figure 11), consistent with research findings from other parts of the world, such as karst regions and the Loess Plateau (Yu et al. 2021; Zhang et al. 2018). The negative correlation between C/N and plant diversity in URNM suggests that lower C/N ratios (Figure 9), which indicate faster organic matter decomposition and higher nutrient availability, support greater plant diversity (Ostrowska and Porebska 2015). SOC is the foundation of soil nutrient cycling, and soils with higher SOC content are usually more fertile, supporting a more diverse plant community (Furey and Tilman 2021; Saint‐Laurent et al. 2014). Lower C/P and N/P ratios indicate relatively higher phosphorus availability (Chen et al. 2018; Liu et al. 2022b), which may promote plant diversity. Furthermore, this study also found that the main soil environmental factors affecting the plant diversity of different life‐form plants differ. For example, C/N and TK were the main factors affecting the plant diversity of the URNM tree layer, while N/P, C/N, and C/P were the main factors affecting the URNM shrub layer. TK was the dominant factor affecting the URNM herb layer (Figure 11). These differences are due to the distinct ecological niches and resource requirements of each plant life form. Trees, which are generally more competitive and less affected by immediate nutrient changes, rely more on the availability of potassium and nitrogen. In contrast, shrubs and herbs, which occupy more disturbed or nutrient‐poor areas, are more sensitive to variations in phosphorus and carbon, as these nutrients are crucial for their faster growth and survival (Laliberté et al. 2014; Zhao et al. 2022).

Our study confirmed that, compared to URNM, TK, SOC, C/N, and N/P were the dominant factors affecting plant diversity in the URMPA and URMPW communities (Figure 11). This predominance is largely attributed to the pronounced human disturbances in URMPA and URMPW, especially management practices such as fertilization and irrigation, which can perturb the original equilibrium of soil environmental factors, causing changes in soil physical and chemical properties as well as in ecological stoichiometry (Guo et al. 2024; Wheeler et al. 2017). Furthermore, additional factors, such as artificial vegetation planting, the proliferation of impervious surfaces within the parks, and the fragmentation of the park landscape into discrete patches, can adversely affect the structure and diversity of the indigenous vegetation community (Cheng et al. 2022; LaPaix and Freedman 2010). These impacts can ultimately modify the coupling relationship between the soil environment and plant diversity. Additionally, this study found that TK was the most important positive dominant factor affecting the diversity of different life‐form plants in URNM, URMPA, and URMPW (Figure 11). The increased dominance of potassium as a key factor affecting plant diversity in URMPA and URMPW highlights the importance of this nutrient in disturbed karst ecosystems. Potassium is essential for plant growth, particularly in enhancing root development and overall plant resilience (Song et al. 2015). The significant role of TK in these disturbed ecosystems underscores its critical contribution to shaping plant community structure, particularly in areas with nutrient limitations, such as karst landscapes (Tan et al. 2006).

### 4.4 Limitations and Future Works

This study has revealed the characteristics and coupling relationships between plant diversity and soil environmental factors in different types of karst urban remnant mountains. These findings provide important scientific evidence for the conservation, maintenance, and sustainable utilization of these ecosystems. However, besides soil environmental factors, other elements such as climate, landscape patterns, topography, and both natural and anthropogenic disturbances also play significant roles in shaping community plant diversity and its distribution patterns (Díaz et al. 2019; Pugnaire et al. 2019; Viljur et al. 2022). The characteristics of soil environmental factors are influenced by a range of factors (Smith et al. 2016), and this study did not conduct a detailed attribution analysis of the changes in soil environmental factors at different slope positions of different types of mountains. Additionally, factors such as the level of tourist disturbance within the park, fertilization frequency, irrigation practices, and the density of internal roads also impact the characteristics and patterns of plant diversity in urban mountain parks (Cheng et al. 2022; Guo et al. 2024; Wheeler et al. 2017). Future research should focus on the comprehensive impact of these factors on the plant diversity of mountain parks to better reveal the mechanisms by which the transformation of urban remnant mountains into mountain parks affects plant diversity.

## 5 Conclusion

We concluded that URNM has higher plant diversity and a richer species composition compared to URMPA and URMPW. In URNM, plant diversity was lowest at the US and highest at the LS, while URMPA and URMPW exhibited an inverse pattern. Soil properties like SBD, TP, and TK were more abundant in URMP. In URNM, the key drivers of plant diversity were the C/N, C/P, and SOC. However, TK was the primary factor influencing diversity in URMPA and URMPW. Overall, the transformation of karst urban remnant mountains into mountain parks had not only simplified the composition of plant species and reduced biodiversity but also changed the characteristics of soil environmental factors and their distribution pattern with plant diversity, thus reshaping their mutual relationship. Future research should focus on the combined effects of soil factors and other elements on plant diversity in mountain parks in order to better elucidate the mechanisms by which the transformation of urban remnant mountains into mountain parks affects plant diversity.

## Author Contributions

**Shujun Liu:** conceptualization (equal), investigation (equal), methodology (equal), software (equal), visualization (equal), writing – original draft (equal). **Zhijie Wang:** conceptualization (equal), funding acquisition (equal), methodology (equal), resources (equal), writing – original draft (equal), writing – review and editing (equal). **Lifei Yu:** methodology (equal), writing – original draft (equal). **Gilbert Kumilamba:** software (equal), writing – review and editing (equal). **Gang Xie:** methodology (equal), software (equal), writing – original draft (equal).

## Conflicts of Interest

The authors declare no conflicts of interest.

## Data Availability

The data for this study are available via the Mendeley Data Repository: https://doi.org/10.17632/7cxzdmpnpn.1.
